# Difference in the Ascending Reticular Activating System Injury Between Mild Traumatic Brain Injury and Cerebral Concussion

**DOI:** 10.1515/tnsci-2019-0017

**Published:** 2019-04-23

**Authors:** Sung Ho Jang, Seong Ho Kim, Han Do Lee

**Affiliations:** 1 Department of Physical Medicine and Rehabilitation, College of Medicine, Yeungnam University, Gyeongsan, South Korea; 2 Department of Neurosurgery, College of Medicine Yeungnam University, Gyeongsan, South Korea

**Keywords:** Mild traumatic brain injury, Cerebral concussion, Ascending reticular activating system, Diffusion tensor tractography, Diffusion tensor imaging

## Abstract

**Introduction:**

We investigated differences in the ascending reticular activating system (ARAS) injuries between patients with mild traumatic brain injury (mTBI) and cerebral concussion by using diffusion tensor tractography (DTT).

**Methods:**

Thirty-one patients with mTBI, 29 patients with concussion, and 30 control subjects were recruited. We used DTT to reconstruct the lower ventral and dorsal ARAS, and the upper ARAS. The fractional anisotropy (FA) value and the fiber number (FN) of the lower ventral and dorsal ARAS, and the upper ARAS were determined.

**Results:**

Significant differences were observed in the FA values of the lower ventral and dorsal ARAS on both sides between the mTBI and control groups and between the concussion and control groups (p < 0.05). The FN value was significantly different in the lower ventral ARAS on both sides between the concussion and control groups and between the mTBI and concussion groups (p < 0.05).

**Conclusion:**

Both the mTBI and concussion patients suffered injuries in the lower ventral and dorsal ARAS, with the concussion patients exhibiting more severe injury in the ventral ARAS than that in the mTBI patients. These results suggest that the terms mTBI and concussion should be used differentially, even though they have used interchangeably for a long time.

## Introduction

Traumatic brain injury (TBI) is a major cause of long-term disability and sequelae in adults [1, 2]. It is classified as mild, moderate, or severe based on the level of severity. Most TBIs are classified as mild TBI (75% to 90% of TBI cases). Definition of mild TBI (mTBI) is based on a duration of loss of consciousness (LOC) of 30 minutes or less, post-traumatic amnesia of 24 hours or less, and a Glasgow coma scale after 30 minutes score of 13~15 [3, 4, 5, 6]. Cerebral concussion is defined as a transient, temporary, and neurological dysfunction caused by trauma that results in temporary LOC for less than six hours. Although mTBI and concussion have different definitions, the two terms have been used interchangeably.

According to the criteria for classification of mTBI and concussion, the duration of LOC is the unique differentiating criterion; therefore, analysis of the ascending reticular activating system (ARAS), which is closely related with LOC, is necessary when investigating the difference between mTBI and concussion. The ARAS is a complex neural network connecting the reticular formation (RF) of the brainstem to the cerebral cortex [7, 8, 9, 10, 11, 12]. Unfortunately, conventional brain magnetic resonance imaging has been limited to demonstrate the analysis of the ARAS.

The introduction of diffusion tensor tractography (DTT), results of which are derived from diffusion tensor imaging (DTI) results, has enabled three-dimensional reconstruction and assessment of the ARAS. Many studies using DTT have demonstrated ARAS injuries in patients with various TBI types [13, 14, 15, 16]. Among these studies, only a few have demonstrated ARAS injury in mTBI [15, 16, 17]. In addition, very little is known about the differences in ARAS injury between mTBI and concussion. We hypothesized that the degree of ARAS injury would be different between mTBI and concussion.

In the current study, we investigated ARAS injuries in mTBI and concussion by using DTT.

## Methods

Sixty-one consecutive patients (28 males, 33 females; mean age 46.71 ± 11.78 years, range 19~65 years) with TBI who had visited the rehabilitation department of a university hospital and 30 right-handed normal subjects (14 males, 16 females; mean age 43.63 ± 10.93 years, range 21~62 years) were recruited for this study. Patients were recruited consecutively according to the following inclusion criteria: 1) age at the time of head trauma: 20~65 years; 2) no history of previous head trauma or neurologic or psychiatric disease; 3) more than one month had elapsed after onset of TBI; 4) no specific brain lesion detected on conventional MRI (T1-weighted, T2-weighted, fluid-attenuated inversion recovery [FLAIR], and T2-weighted gradient recall echo images). 5) LOC at the time of injury without a lucid interval. The study was conducted retrospectively, and the study protocol was approved by the institutional review board of a university hospital.

The patients with TBI that met the inclusion criteria were classified according to LOC: 31 (50.81%) of the 61 TBI patients (15 males, 16 females; mean age 45.85 ± 10.03 years) belonged to the mTBI group (*i.e*., LOC for less than 30 minutes) and 29 (49.18%, mean age 47.57 ± 13.43 years) belonged to the concussion group (*i.e*., LOC for less than 6 hours and with headache and memory problem symptoms after trauma) [18, 19]. Demographic data for the mTBI and concussion groups are summarized in table 1. Demographic characteristics did not differ between the mTBI and concussion groups (*p* > 0.05).

**Table 1:** Demographic data of the mild traumatic brain injury and concussion groups

Variables	mTBI group	Concussion group
Patients, n (%)	31 (50.81%)	29 (49.18%)
Age (year)	45.85 (10.03)	47.57 (13.43)
Sex, male/female	15/16	13/17
Duration to DTI (months)	3.03 (6.02)	3.85 (6.32)
LOC (minutes / hours)	7.11 (6.47) (minutes)	3.13 (1.80) (hours)

### Diffusion tensor imaging and tractography

DTI data were acquired at an average of 3.35 ± 6.08 months after the onset of TBI by using a 1.5 T Philips Gyroscan Intera (Hoffman-LaRoche, Best, Netherlands) with 32 non-collinear diffusion sensitizing gradients by performing single-shot echo-planar imaging. For each of the 32 non-collinear diffusion sensitizing gradients, 67 contiguous slices were acquired parallel to the anterior commissure–posterior commissure line. Imaging parameters were as follows: acquisition matrix = 96 × 96; reconstructed matrix = 192 × 192; field of view = 221 mm × 221 mm; TR = 10,726 ms; TE = 76 ms; parallel imaging reduction factor (SENSE factor) = 2; EPI factor = 49; b = 1000 s/mm^2^; NEX = 1; and slice thickness = 2.5 mm with no gap (acquired voxel size 1.25 mm × 1.25 mm × 2.5 mm). The Oxford Centre for Functional Magnetic Resonance Imaging of the Brain (FMRIB) Software Library (www.fmrib.ox.ac. uk/fsl) was used for analysis of DTI data. Affine multi-scale two-dimensional registration was used for correction of head motion effects and image distortion due to eddy currents. FMRIB Diffusion software with the routines option (0.5 mm step lengths, 5000 streamline samples, curvature thresholds = 0.2) was used for fiber tracking. Two portions of the lower ARAS reconstructed by selecting fibers passing through the following regions of interest (ROIs): the dorsal lower ARAS – the seed ROI placed on the pontine reticular formation (RF), and the target ROI placed on the intralaminar thalamic nucleus (ILN); the ventral lower ARAS – the seed ROI placed on the pontine RF, the target ROI placed on the hypothalamus [15, 20]; and the upper ARAS – the seed ROI placed on the LN of the thalamus at the level of the inter-commissural plane between the anterior and posterior commissures [21]. Out of 5000 samples generated from the seed voxel, contact results were visualized at a threshold for the lower dorsal and ventral ARAS at a minimum of 2 contacts and for upper neural connectivity of the thalamic ILN of 15 contacts, and were streamlined through each voxel for analysis.

Values represent mean (±standard deviation). mTBI: mild traumatic brain injury, DTI: diffusion tensor imaging, LOC: loss of consciousness

**Figure 1:**
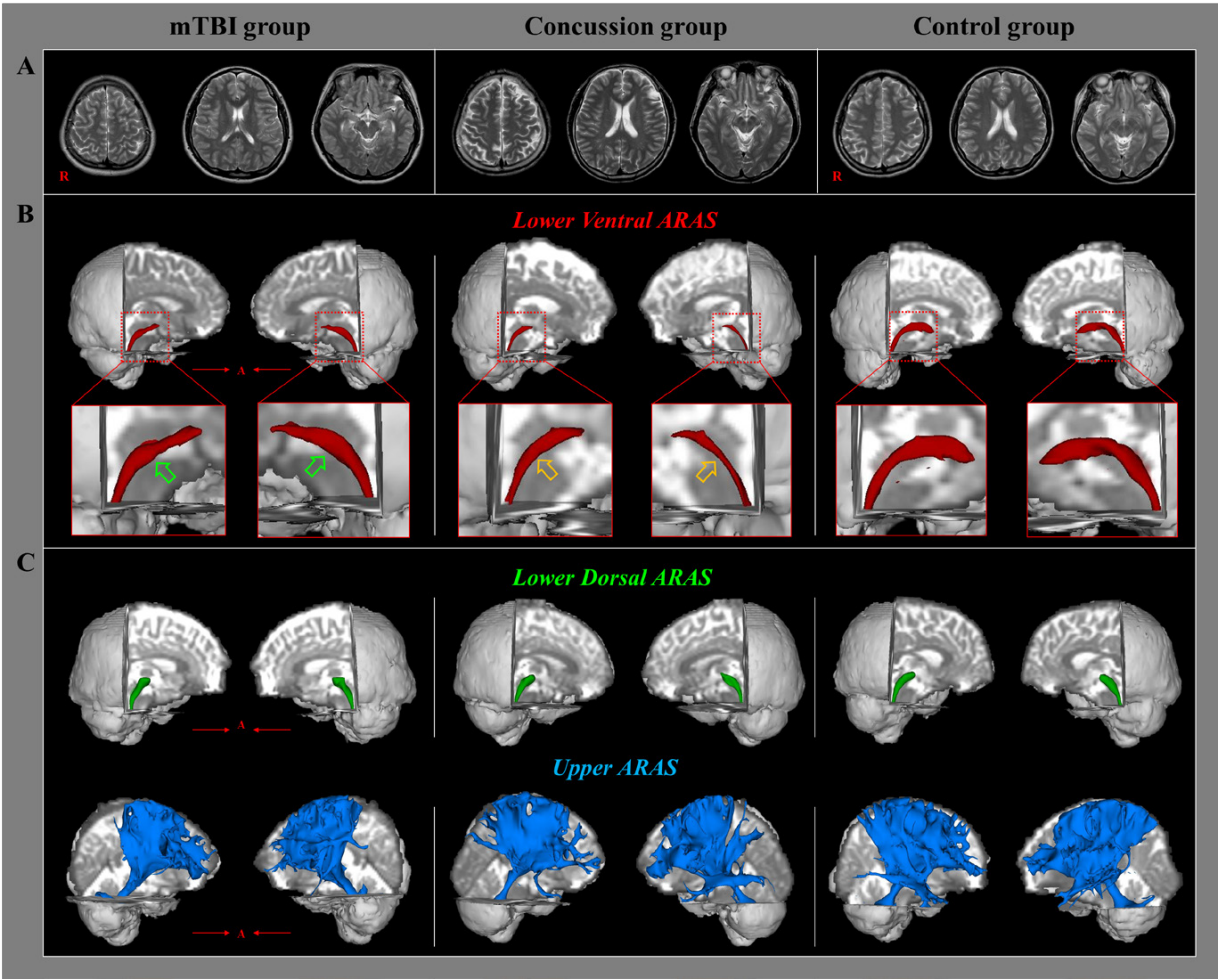
(A) T2-weighted brain magnetic resonance images at the time of diffusion tensor imaging scanning in representative patients with mild traumatic brain injury (mTBI; 51-year-old female) and concussion (48-year-old female) and in a control subject (50-year-old female). (B) Results of diffusion tensor tractography (DTT) for the lower ventral ascending reticular activating system (ARAS): the lower ventral ARAS of the mTBI (green arrows) and concussion (orange arrows) groups are thinner compared with the control group and that of the concussion group is thinner than that in the mTBI group (orange arrows). (C) Results of DTT for lower dorsal and upper ARAS show similar findings among the three groups.

The fractional anisotropy (FA) values and fiber number (FN) of the lower and upper ARAS were also determined.

### Statistical analysis

SPSS software (v. 18.0; SPSS, Chicago, IL, USA) was used for data analysis. One-way analysis of variance (ANOVA) was performed for determination of significant differences in the lower (ventral and dorsal) and upper ARAS for each of the DTT parameters (*i.e*., FA and FN) between the mTBI, concussion, and control groups. When using ANOVA, if a significant difference was detected among the three groups, a least significant difference *post hoc* test was performed to determine the significance of differences in the DTT parameters among the three groups. Statistical significance was accepted for *p* values of < 0.05.

## Results

The results of DTT parameters for the lower (ventral and dorsal) and upper ARAS in the three groups are summarized in table 2. Significant differences were observed in the FA values of the lower ventral and dorsal ARAS (both sides) between the mTBI and control groups and between the concussion and control groups (*p* < 0.05). However, a significant difference was not observed in the FA values of the lower ventral and dorsal ARAS (both sides) between the mTBI and concussion groups (*p* > 0.05). The FN value was significantly different in the lower ventral ARAS (both sides) between the concussion and control groups and between the mTBI and concussion groups (*p* < 0.05). However, we did not detect significant differences in the FN value of lower ventral ARAS (both sides) between the mTBI and control groups, or of the lower dorsal ARAS (both sides) between each of the three groups (*p* > 0.05). In addition, no significant differences were detected in the FA and FN values in upper ARAS (both sides) between each of the three groups (*p* > 0.05).

**Table 2:** Comparison of diffusion tensor tractography parameters for the ascending reticular activating system between the mild traumatic brain injury and concussion groups

		Right			Left		
		FA	FN		FA		FN
				Lower ventral ARAS			
	mTBI group	0.37 ± 0.04	293.68 ± 117.06		0.37 ± 0.03		301.72 ± 114.30
	Concussion group	0.37 ± 0.06	221.73 ± 121.89		0.36 ± 0.06		226.61 ± 59.99
	Control group	0.41 ± 0.03	343.71 ± 67.62		0.41 ± 0.05		346.13 ± 107.25
				Lower dorsal ARAS			
	mTBI group	0.39 ± 0.04	356.06 ± 163.36		0.39 ± 0.02		375.66 ± 182.10
	Concussion group	0.39 ± 0.06	339.27 ± 163.71		0.39 ± 0.04		356.10 ± 0.04
	Control group	0.42 ± 0.03	382.03 ± 141.58		0.42 ± 0.03		412.03 ± 145.11
				Upper ARAS			
	mTBI group	0.36 ± 0.02	10500.41 ± 4617.71		0.37 ± 0.02		11153.43 ± 4241.51
	Concussion group	0.35 ± 0.02	15111.86 ± 4240.95		0.35 ± 0.02		11378.35 ± 5060.18
	Control group	0.36 ± 0.01	10311.16 ± 3240.42		0.36		10282.91 ± 3663.50
				*P* – value			
				Lower ventral ARAS			
		*Rt*	*Lt*			*Rt*	*Lt*
*FA*	mTBI – Control	0.01*	0.01*	FN	mTBI – Control	*0.09*	*0.08*
	Concussion – Control	0.01*	0.00*		Concussion – Control	0.01*	0.01*
	mTBI - Concussion	0.61*	0.58*		mTBI - Concussion	0.01*	0.05*
				Lower dorsal ARAS			
		*Rt*	*Lt*			*Rt*	*Lt*
*FA*	mTBI – Control	0.05*	0.01*	FN	mTBI – Control	*0.69*	*0.09*
	Concussion – Control	0.04*	0.01*		Concussion – Control	0.35*	0.09*
	mTBI - Concussion	0.86*	0.44*		mTBI - Concussion	*0.59*	*0.99*
				Upper ARAS			
		*Rt*	*Lt*			*Rt*	*Lt*
*FA*	mTBI – Control	0.69*	0.15*	FN	mTBI – Control	*0.30*	*0.44*
	Concussion – Control	0.08*	0.63*		Concussion – Control	0.48*	0.34*
	mTBI - Concussion	0.13*	0.36*		mTBI - Concussion	*0.31*	*0.84*

ARAS: ascending reticular activating system, FA: fractional anisotropy, FN: fiber number, mTBI: mild traumatic brain injury, Rt: right, Lt: left, means ± standard deviation, * *p* < 0.05.

## Discussion

In the current study, we investigated differences among three parts of the ARAS between mTBI and concussion. Our results were as follow: 1) the mTBI group – a decrement in FA value was detected in the lower ventral and dorsal ARAS compared with the control group; 2) the concussion group – decrements in FA and FN values were detected in the lower ventral ARAS compared with the control group, and a decrement in FA value was observed in the lower dorsal ARAS compared with the control group; and 3) comparison between the mTBI and control groups – a decrement in FN value was detected only in the lower ventral ARAS in the concussion group.

The FA and FN values have been the most commonly been used parameters among DTI parameters when evaluating the state of a neural tract [22, 23, 24]. The FA value indicates the degree of directionality of water diffusion and indicates white matter organization. It reflects the fiber density axonal diameter, and myelination in white matter [22, 23]. The FN value is determined by counting the number of voxels in a neural tract, and it reflects the total number of fibers in a neural tract [22, 23, 24]. Therefore, a decrement in FA and/or FN values indicates an injury of a neural tract [22, 23, 24]. The decrements of FA and/or FN in the lower ventral and dorsal ARAS in the mTBI and concussion groups compared with the control group suggests injury of the ventral and dorsal ARAS. Because no definite brain lesions were observed on conventional brain MRI of the patient group, traumatic axonal injury, which indicates tearing of axons due to indirect shearing forces during acceleration, deceleration, and rotation of the brain, or the result of direct head trauma, appears to be the most likely pathogenetic mechanism for injury of the ventral and dorsal ARAS in the mTBI and concussion groups [25, 26].

Regarding the difference between the mTBI and concussion groups, the FN value was lower in the concussion group than in the mTBI group. This result means that the concussion group exhibited greater injury severity in the ventral ARAS than that in the mTBI group. Previous studies have reported associations of narcolepsy, fatigue, and hypersomnia with injury of the lower ventral ARAS [15, 16, 17]. On that basis, our results suggest that the concussion subjects may exhibit more clinical symptoms such as narcolepsy, fatigue, and hypersomnia due to more severe injury of lower ventral ARAS compared with the mTBI group. However, because this study was conducted retrospectively, we were unable to confirm a correlation between injury of lower ventral ARAS and clinical symptoms. Prospective studies to elucidate this correlation should be encouraged.

With regard to the upper ARAS, there was no significant difference detected between the mTBI and concussion groups, suggesting that upper ARAS injury severity was not different between the mTBI and concussion groups. The upper ARAS, including the prefrontal cortex, basal forebrain, and parietal lobe, is reported to be important for awareness [27, 28, 29, 30]. The measurement of DTT parameters for an entire, large neural tract like the upper ARAS can lead to false negative results because an axonal lesion may be focal or minor compared with that in the whole neural tract [31]. Therefore, further studies with detailed analysis of a divided upper ARAS are warranted.

Since the introduction of DTI, several studies have reported on patients with mTBI [15, 16, 17]. However, no previous study has reported on DTI differences between mTBI and concussion. As a result, to the best of our knowledge, this is the first study to demonstrate a difference in the ARAS injuries between mTBI and concussion. However, several limitations of this study should be considered. First, the concussion group in this study included patients with more severe concussion than that among all concussion patients because we classified patients with an LOC less than 30 minutes as members of the mTBI group. Second, since patients were recruited from those who visited the rehabilitation department of a university hospital, there was a possibility that patients with severe clinical features might be included in this study, as compared to a more general population of patients with TBI. Lastly, although DTT is a powerful anatomic imaging tool that can demonstrate gross fiber architecture, DTT may underestimate the fiber tracts due to regions of fiber complexity and crossing, which can prevent full reflection of the underlying fiber architecture [32].

In conclusion, we observed that both mTBI and concussion patients exhibited injuries in the lower ventral and dorsal ARAS, with concussion patients exhibiting more severe injury in the ventral ARAS than that in mTBI patients. Thus, our results suggest that the terms mTBI and concussion should be used differentially, even though the two terms have used interchangeably for a long time.
